# A randomised phase II study of weekly paclitaxel or vinorelbine in combination with cisplatin against inoperable non-small-cell lung cancer previously untreated

**DOI:** 10.1038/sj.bjc.6601526

**Published:** 2004-01-20

**Authors:** Y-M Chen, R-P Perng, J-F Shih, Y-C Lee, C-S Lee, C-M Tsai, J Whang-Peng

**Affiliations:** 1 Chest Department, Taipei Veterans General Hospital, Taipei, Taiwan; 2 National Yang-Ming University, N0155, Sec. 2, Li-Long Street, Taipei 112, Taiwan; 3 Division of Cancer Research, National Health Research Institute, Taipei, Taiwan

**Keywords:** cisplatin, non-small-cell lung cancer, paclitaxel, vinorelbine

## Abstract

Phase II studies have suggested that weekly paclitaxel has a higher response rate and better toxicity profile than the conventional schedule of once every 3 or 4 weeks. Our aim was to evaluate the efficacy of weekly paclitaxel plus cisplatin (PC) *vs* vinorelbine plus cisplatin (VC) in chemonaïve non-small-cell lung cancer (NSCLC) patients. From October 2000 to May 2002, 140 patients were enrolled. The treatment dose was P 66 mg m^−2^ intravenous infusion (i.v.) on days 1, 8, and 15, and C 60 mg m^−2^ i.v. on day 15, or V 23 mg m^−2^ i.v. on days 1, 8, and 15, and C 60 mg m^−2^ i.v. on day 15, every 4 weeks. In all, 281 cycles of PC and 307 cycles of VC were given to the patients in the PC and VC arms, respectively. There were 26 partial responses and one complete response (overall 38.6%) in the PC arm, and no complete responses, but 27 partial responses (overall 38.6%) in the VC arm. Myelosuppression was more common in the VC arm (*P*<0.001). Peripheral neuropathy and myalgia were significantly more common in the PC arm (*P*<0.001). The median time to disease progression was 6 months in the PC arm and 8.4 months in the VC arm (*P*=0.0344). The median survival time was 11.7 months in the PC arm and 15.4 months in the VC arm (*P*=0.297). We concluded that weekly PC is not suggested for NSCLC patients due to the relatively shorter progression-free survival and more common nonhaematological toxicities.

Lung cancer is the most common cancer in Taiwan, and throughout the world (Parkin *et al*, 1999). The reported 5-year survival was 14% in the US and 8% in Europe (Parkin *et al*, 1999). The high proportion of disseminated disease and poor prognoses in these patients has encouraged the decades-long continued efforts at developing new chemotherapeutic options.

In the past decade, cisplatin in combination with new anticancer drugs has been found to increase effectively, although modestly, patient survival in randomised trials and/or meta-analyses, when compared with cisplatin plus conventional anticancer drugs (Grilli *et al*, 1993; Le Chevalier *et al*, 1994; NSCLC collaborative group, 1995; Bonomi *et al*, 2000; Baggstrom *et al*, 2002; Raftopoulos *et al*, 2002; Schiller *et al*, 2002; Yana *et al*, 2002). Among these new anticancer drugs, vinorelbine was the first drug to show better survival, when in combination with cisplatin, than conventional anticancer drugs in combination with cisplatin, in a randomised phase III study (Le Chevalier *et al*, 1994). However, which new anticancer drugs in combination with cisplatin will have the best efficacy, in terms of longer survival and better toxicity profiles, is still unknown (Schiller *et al*, 2002).

In spite of the fact that a phase III randomised trial showed better survival with paclitaxel plus cisplatin, when comparing etoposide with cisplatin (Bonomi *et al*, 2000), many studies have not demonstrated better survival with paclitaxel plus cisplatin or carboplatin, when compared with conventional chemotherapy (Giaccone *et al*, 1998; Gatzemeier *et al*, 2000; Raftopoulos *et al*, 2002). The dosage and schedule of the paclitaxel used varied widely among the different studies and, thus, probably had different therapeutic effects on the patients. In contrast to the conventional paclitaxel treatment schedule of once every 3 or 4 weeks, weekly paclitaxel treatment was found to have a higher response rate and better toxicity profiles in phase II trials of both chemonaïve patients and patients who had failed previous chemotherapy (Akerley *et al*, 1998; Belani, 2001; Chang *et al*, 2001; Koumakis *et al*, 2002). However, there has been no report of randomised phase II or III trials comparing weekly paclitaxel with other new anticancer drugs. There also has not been a report on the weekly dosage of paclitaxel that could be safely used with Asian patients, especially when combining cisplatin treatment. Thus, we conducted a phase II randomised trial using weekly paclitaxel or vinorelbine plus cisplatin treatment in inoperable, chemonaïve non-small-cell lung cancer (NSCLC) patients to investigate the efficacy of these regimens.

## PATIENTS AND METHODS

### Patients

This study was conducted according to existing rules for good clinical practice, and the protocol was approved by the local ethics committee. All patients signed informed consent before entry into the study. Those eligible for entry into the study were patients with a cytologic or histologic diagnosis of NSCLC; stage IIIb, IV, or recurrence after surgical treatment; aged 18–80 years; no prior chemotherapy, immunotherapy, or radiotherapy; a performance status of 0–2 on the World Health Organization (WHO) scale; bidimensionally measurable disease; and adequate bone marrow reserve with a white blood cell count ⩾4000 mm^−3^, platelets ⩾100 000 mm^−3^, and hemoglobin ⩾10 g/dl. Patients with signs or symptoms of brain metastases; inadequate liver function (bilirubin >1.5 times and ALT/AST >3 times upper limit normal); or inadequate renal function with creatinine >2.0 mg/dl were excluded from the study.

Initial work-up included the documentation of the patient's history, a physical examination, and a performance score. A complete blood cell count, urinalysis, serum biochemistry profile, ECG, chest roentgenography, whole body bone scan, brain CT scan, and chest CT scan (including liver and adrenal glands) were also performed.

### Study design

Eligible patients were randomised into either the weekly paclitaxel plus cisplatin regimen (PC arm) or vinorelbine plus cisplatin regimen (VC arm) by a statistical office not involved in the trial. Paclitaxel or vinorelbine was given on days 1, 8, and 15 of every 4 weeks. Cisplatin 60 mg m^−2^ intravenous infusion (i.v.) was given on day 15. Initially, nine patients from each treatment arm entered a rapid dose escalation course to determine the weekly dosage of paclitaxel (escalated from 60 to 66, 72, … mg m^−2^) and vinorelbine (escalated from 20 to 23, 26, … mg m^−2^) that would result in the highest dose intensity. The optimal treatment dose was found to be paclitaxel 66 mg m^−2^ i.v. on days 1, 8 and 15 and cisplatin 60 mg m^−2^ i.v. on day 15, or vinorelbine 23 mg m^−2^ i.v. on days 1, 8, and 15 and cisplatin 60 mg m^−2^ i.v. on day 15, every 4 weeks. All patients in the PC arm received dexamethasone (10 mg i.v.), cimetidine (300 mg i.v.), diphenhydramine (50 mg i.v.), and metoclopramide (20 mg i.v.) before paclitaxel administration. All patients in the VC arm received dexamethasone (10 mg i.v.) and metoclopramide (20 mg i.v.) before vinorelbine administration. In addition to the above-mentioned premedications, granisetron and adequate hydration with half saline was also given on day 15, when cisplatin treatment was given.

With regard to dose modifications within a cycle, the dose of paclitaxel or vinorelbine was reduced by 50% if the absolute neutrophil count (ANC) was from 1.5 to 1.0 × 10^9^ l^−1^ and/or the platelet count was 99 to 75 × 10^9^ l^−1^on the day of the scheduled chemotherapy. Treatment was omitted if the ANC was less than 1.0 × 10^9^ l^−1^ or the platelet count was less than 75 × 10^9^ l^−1^. For day 15 treatment, CBC was checked every other day after day 15 if the data on day 15 showed an ANC less than 1.0 × 10^9^ l^−1^ or a platelet count less than 75 × 10^9^/ l^−1^, and treatment was given once if the ANC was above 1.0 × 10^9^ l^−1^ and the platelet count was above 75 × 10^9^ l^−1^ (cisplatin was given at 75% of scheduled dose if the ANC was 1–1.5 × 10^9^ l^−1^ and/or the platelet count was 99–75 × 10^9^ l^−1^). For dose adjustments in the subsequent cycle, a 25% reduction in paclitaxel or vinorelbine was instituted when the patient suffered from grade 4 neutropenia or thrombocytopenia. Subsequent dose escalation to the original dosage was allowed providing the patient tolerated the doses given at the 75% level. For nonhaematological toxicities, paclitaxel or vinorelbine, and cisplatin were reduced to a 75% dose if there were grade 3 toxicities, and the patient went off-study with grade 4 toxicities, excluding those due to nausea/vomiting or alopecia. Patients went off-study if they suffered from grade 3 or worse neuropathy.

After maximal effective chemotherapy (chemotherapy given to a degree that measurable lesion(s) could not further decrease in size), radiotherapy was given to all stage IIIb patients, excluding those with malignant pleural effusion.

### Response evaluation

A complete blood cell count was repeated before every injection. Serum biochemistry was performed before every course of chemotherapy, and during the course, if clinically indicated. The Lung Cancer Symptom Scale was recorded before every course of chemotherapy and when the patient completed or went off the study (Hollen and Gralla, 1996; Lutz *et al*, 1997).

Response and study drug-related toxicities were evaluated according to WHO criteria (World Health Organization, 1979). Patient responses were reevaluated after every two cycles. In responding patients and patients with stable disease, a maximum of six cycles of chemotherapy was given. Those patients whose tumours progressed were taken off study as soon as this finding was documented clinically and/or radiographically. All adverse events, whether thought to be due to chemotherapy or not, were recorded.

### Statistical methodology

This study was designed to enroll 68 qualified patients in each arm. This calculation assumes that the true response probability for the best treatment is 10% better than that for the others. Assume that the smaller treatment response rate was 25% and the higher treatment response rate was 35%, with a power of 0.9 and a *P-*value of 0.05, and that each treatment group requires 68 qualified patients (Simon *et al*, 1985).

The response rate and survival were all analysed with an intention-to-treat principle. The overall survival and time-to-disease progression were analysed using the Kaplan–Meier estimation method. Time-to-disease progression was calculated from the date of initiation of treatment to the date of disease progression or death. If disease progression had not occurred by the time of this analysis, progression-free survival was considered censored at the time of the last follow-up. Survival time was measured from the date of initiation of treatment to the date of death. If death had not occurred, survival time was considered censored at the last follow-up time. All comparisons of clinical characteristics, response rates, and toxicity incidences were performed using the ANOVA test. For the statistical analysis of the Lung Cancer Symptom Scale, the Mann–Whitney test was used for a comparison of the two arms of treatment, and the Wilcoxon signed-ranks test was used for comparison before and after treatment.

## RESULTS

### Patient characteristics

From October 2000 to May 2002, 140 patients were entered and randomised into this study, including 70 patients in each arm. Patient ages ranged from 23 to 80 years, with mean ages of 64.9 years in the PC arm and 64.8 years in the VC arm. The clinical characteristics of these patients are listed in Table 1

**Table 1:** Patient characteristics

	**Patient number (%)**		
**Variable**	**Paclitaxel+cisplatin**	**Vinorelbine+cisplatin**	***P-*value^a^**
Patient number	70	70	0.058
Male	56	46	
Female	14	24	
Age: mean (range)	64.9 (37–0)	64.8 (23–0)	0.97
WHO performance status			
0	13 (18.6)	8 (11.4)	0.412
1	26 (37.1)	29 (41.4)	
2	31 (44.3)	33 (47.1)	
Stage	19 (27.1)	16 (22.9)	0.54
IIIB			
IV	46 (65.7)	48 (68.6)	
Recurrence after surgery	5 (7.1)	6 (8.6)	
Histology			
Adenocarcinoma	46 (65.7)	39 (55.7)	0.407
Squamous cell carcinoma	10 (14.3)	16 (22.9)	
NSCLC, type not specified	14 (20)	15 (21.4)	

a There was no statistical difference in patient clinical characteristics between the two treatment groups.

. There was no statistical difference in the clinical characteristics of the two arms of treatment in terms of sex, age, stage, performance status, histology, and number of metastases in stage IV patients. All patients were assessable for toxicity profile and 132 patients were evaluable for treatment response.

### Treatment received

The 70 patients in the PC arm received a total of 281 cycles of treatment with a median of four cycles (mean 4.01, range 1–6), and the 70 patients in the VC arm underwent a total of 307 cycles of treatment with a median of five cycles (mean 4.39, range 1–6). The mean percentage of dose administered in the PC arm was 95.3% of the scheduled paclitaxel dose on day 1, 90% on day 8, and 85.2% on day 15, and 88.9% of the scheduled cisplatin dose on day 15. The mean percentage of dose administered in the VC arm was 93.1% of the scheduled vinorelbine dose on day 1, 95.3% on day 8, and 83.3% on day 15, and 87.6% of the scheduled cisplatin dose on day 15 (Table 2

**Table 2:** Dose intensity of paclitaxel plus cisplatin treatment *vs* vinorelbine plus cisplatin treatment

		**Full dose injections**	**75% dose injections**	**50% dose injections**	**Omit or off-study**	**% dose administered**
*Paclitaxel+cisplatin*^a^						
Paclitaxel	Day 1	237	35	9	0	95.3
	Day 8	223	32	12	14	90
	Day 15	201	36	23	21	85.2
Cisplatin	Day 15	231	21	6	23	88.9
*Vinorelbine+cisplatin*^a^						
Vinorelbine	Day 1	252	25	30	0	93.1
	Day 8	272	22	8	0	95.3
	Day 15	204	37	48	18	83.3
Cisplatin	Day 15	230	38	21	18	87.6

a A total of 281 cycles in the paclitaxel plus cisplatin arm and 307 cycles in the vinorelbine plus cisplatin arm.

). Asthenia and myalgia were the major causes of dose reduction in patients receiving PC treatment. In contrast, neutropenia accounted for the majority of dose reductions in patients undergoing VC treatment. Among these 140 patients, 24 were stage IIIb without malignant effusion (15 patients in the PC arm, nine patients in the VC arm). A total of 14 patients received radiotherapy after four to six cycles of chemotherapy (eight patients in the PC arm, six patients in the VC arm), five patients were not eligible for radiotherapy because of inadequate pulmonary reserve (three patients in the PC arm, two patients in the VC arm), one patient in the PC arm had early disease progression, and four patients refused radiotherapy (three patients in the PC arm, one patient in the VC arm).

### Response

After two cycles of treatment, one patient achieved a complete response and 26 patients achieved a partial response in the PC arm, and 27 patients achieved a partial response in the VC arm. Both arms had an overall response rate of 38.6% (95% confidence interval, 29.3–47.9%, Table 3

**Table 3:** Overall response rate

	**Number of Patients (%)**	
	**Paclitaxel+cisplatin (*n*=70)**	**Vinorelbine+cisplatin (*n*=70)**
Overall response	27 (38.6)	27 (38.6)
Complete response	1 (1.4)	0
Partial response	26 (37.2)	27 (38.6)
Stable disease	25 (35.7)	32 (45.7)
Progressive disease	14 (20)	7 (10)
Unevaluable	4 (5.7)	4 (5.7)

The 95% confidence interval of the overall response rate was the same in both arms: 29.3–47.9%.

). In all, 10 of 19 stage IIIb patients in the PC arm and six of 16 stage IIIb patients in the VC arm had a partial response to the treatment. The response rate did not correlate with the patients’ performance status and staging in either arm.

**Figure 1:**
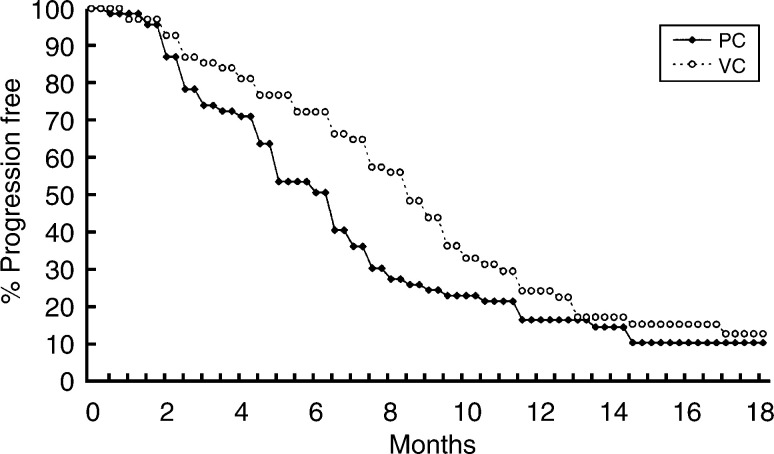
Time to disease progression of 140 NSCLC patients treated with cisplatin plus weekly paclitaxel (PC) or vinorelbine (VC). The median time to disease progression was 6 months in the PC arm, and 8.4 months in the VC arm (*P*=0.0344).

**Figure 2:**
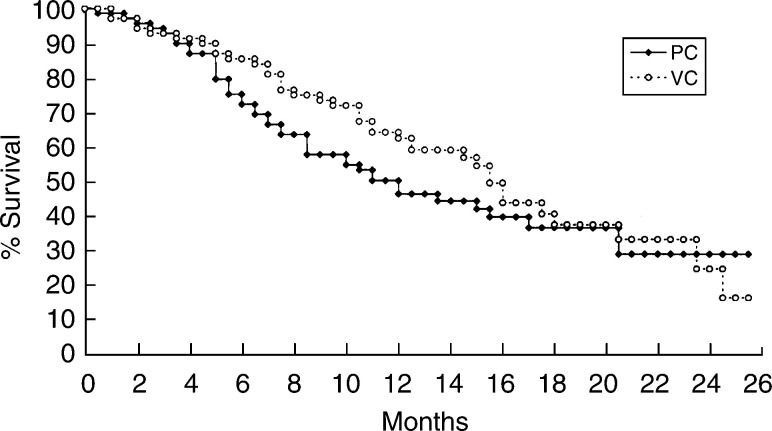
Survival curve of 140 NSCLC patients treated with cisplatin plus weekly paclitaxel (PC) or vinorelbine (VC). The median survival time was 11.7 months in the PC arm and 15.4 months in the VC arm after a median follow-up time of 18 months (*P*=0.297).

After a median follow-up time of 18 months, 42 patients in the PC arm and 38 patients in the VC arm had died. The median time to disease progression was 6 months in the PC arm, and 8.4 months in the VC arm (Figure 1

, *P*=0.0344). The median survival time was 11.7 months in the PC arm (95% confidence interval 7.8–15.6 months) and 15.4 months in the VC arm (95% confidence interval 13.9–16.8 months) (*P*=0.297, Figure 2

). The 1-year survival rate was 46.3 and 60.9%, respectively (*P*=0.12).

### Toxicity

All patients enrolled into the study were eligible for toxicity evaluation. The toxicities were generally mild and reversible, except for some patients who were left with grade 1 peripheral neuropathy that lasted for several months. The main toxicities were haematological (leucopenia and neutropenia) in the VC arm of treatment (Table 4

**Table 4:** Haematological toxicity per patient (worst of any course, *n*=70 in both arms)

	**Number of patients (%)**									
**Toxicitygrade**	**Paclitaxel+cisplatin**					**Vinorelbine+cisplatina**				
	0	1	2	3	4	0	1	2	3	4
Leukopenia^b^	37 (52.9)	15 (21.4)	15 (21.4)	3 (4.3)	0	15 (21.4)	16 (22.9)	19 (27.1)	18 (25.7)	2 (2.9)
Neutropenia^b^	39 (55.7)	13 (18.6)	11 (15.7)	4 (5.7)	3 (4.3)	13 (18.6)	9 (12.8)	11 (15.7)	24 (34.2)	13 (18.6)
Anaemia	4 (5.7)	20 (28.6)	39 (55.7)	7 (10)	0	2 (2.9)	18 (25.7)	40 (57.1)	9 (12.9)	1 (1.4)
Thrombocytopenia	63 (90)	5 (7.1)	1 (1.4)	1 (1.4)	0	67 (95.7)	2 (2.9)	1 (1.4)	0	0

^a^Febrile neutropenia occurred in two patients treated with vinorelbine and cisplatin and one patient died in spite of antibiotics and G-CSF treatment.

b ANOVA test with *P* <0.001.

), and peripheral neuropathy and myalgia in the PC arm of treatment (Table 5

**Table 5:** Nonhematological toxicity per patient (worst of any course, *n*=70 in both arm)

	**Number of patients (%)**									
	**Paclitaxel + cisplatin**					**Vinorelbine + cisplatin**				
**Toxicitygrade**	**0**	**1**	**2**	**3**	**4**	**0**	**1**	**2**	**3**	**4**
Nausea	47 (67.1)	8 (11.4)	15 (21.4)	0	0	42 (60)	15 (21.4)	13 (18.6)	0	0
Vomiting	50 (71.4)	8 (11.4)	11 (15.7)	1 (1.4)	0	53 (75.7)	6 (8.6)	10 (14.3)	1 (1.4)	0
Peripheral neuropathy^a^	8 (11.4)	22 (31.4)	37 (52.9)	3 (4.3)	0	21 (30)	30 (42.9)	19 (27.1)	0	0
Asthenia	7 (10)	13 (18.6)	37 (52.9)	13 (18.6)	0	6 (8.6)	11 (15.7)	47 (67.1)	5 (7.1)	1 (1.4)
Myalgia^a^	24 (34.3)	23 (32.9)	21 (30)	2 (2.9)	0	45 (64.3)	21 (30)	4 (5.7)	0	0
Alopecia^a^	10 (14.3)	33 (47.1)	27 (38.6)	0	0	13 (18.6)	54 (77.1)	3 (4.3)	0	0

a ANOVA test with *P* <0.001. Other toxicities included: six Gr. 1 and two Gr. 2 mucositis, five Gr. 1 constipation, one Gr. 1 and three Gr. 2 pulmonary, one Gr. 3 renal, two Gr. 1 and two Gr. 2 skin rash, one Gr. 2 and one Gr. 3 diarrhoea in paclitaxel + cisplatin arm, four Gr. 1 and four Gr. 2 mucositis, three Gr. 1 pulmonary, one Gr. 1 and one Gr. 2 constipation, one Gr. 2 and one and Gr. 3 diarrhoea, and one hepatitis B reactivation. Gr.=grade; P=paclitaxel; C=cisplatin.

). Febrile neutropenia occurred in two patients treated with VC, and one of them died in spite of antibiotics and G-CSF treatment. This patient accounted for the only toxic death in this study. The incidence of WHO grade 3 or 4 haematological toxicity per patient was: leucopenia 4.3%, neutropenia 10%, anaemia 10%, and thrombocytopenia 1.4% in the PC arm, and leucopenia 28.6%, neutropenia 52.9%, anaemia 14.3%, and thrombocytopenia 0% in the VC arm. The incidence of grade 3 or 4 leucopenia and neutropenia was significantly higher in the VC arm (*P*<0.001). In addition, three patients in the VC arm needed G-CSF support during their treatment course, while no patient in the PC arm needed this support. The incidence of WHO grade 2 or 3 peripheral neuropathy per patient was 57.1% in the PC arm and 27.1% in the VC arm (*P*<0.001). The incidence of WHO grade 2 or 3 myalgia per patient was 32.9% in the PC arm and 5.7% in the VC arm (*P*<0.001). Asthenia was noted in both arms, but was more severe in patients receiving PC treatment. Grades 3–4 asthenia occurred in 13 patients (18.6%) in the PC arm and in six patients (8.6%) in the VC arm. Several other mild and tolerable nonhaematological toxicities were present in both arms (Table 5). A packed RBC transfusion was needed in 39 patients treated with PC, with a total of 207 units, and in 40 patients treated with VC, with a total of 233 units.

### Quality of life

In all, 124 patients (62 patients in each arm) completed the baseline Lung Cancer Symptom Scale questionnaire, and after two cycles of treatment and/or after going off study. The results of the completed Lung Cancer Symptom Scale showed that there was no statistically significant difference in the scales between the PC and VC arms, either before or two cycles after treatment, or when the patient went off the study. This held true whether scored by the patients (nine items) or by the observers (six items), and included the categories of loss of appetite, fatigue, cough, dyspnea, haemoptysis, pain, disease severity, daily activity, and quality of life; however, loss of appetite and pain were worse after two cycles of treatment in the PC arm (Table 6

**Table 6:** Changes in Lung Cancer Symptom Scales in 124 patients who completed the questionnaires^a^

	**PC arm (mean±s.d.)**			**VC arm (mean±s.d.)**		
	**Baseline**	**After C2**	**Off study**	**Baseline**	**After C2**	**Off study**
Appetite loss						
Patient	84±17	75±16	71±16	87±15	76±11	72±14
Observer	87±20	75±21^b^	71±23	92±15	84±17	75±20
Fatigue						
Patient	81±17	70±14	66±15	84±15	72±12	66±14
Observer	84±21	73±20	66±21	88±18	79±17	68±19
Cough						
Patient	83±16	78±15	75±15	83±14	77±14	76±17
Observer	89±17	85±17	82±19	87±16	82±19	78±19
Dyspnea						
Patient	77±15	71±13	68±12	79±14	72±12	68±12
Observer	82±16	75±17	72±16	84±15	79±15	73±16
Hemoptysis						
Patient	96±12	96±10	96±10	99±7	98±8	98±8
Observer	96±13	97±8	98±8	97±13	98±7	98±8
Pain						
Patient	86±19	73±17^b^	71±18	87±17	80±16	75±16
Observer	88±21	76±20^b^	71±22^b^	90±18	86±17	81±18
Disease severity, patient	80±16	71±15	67±15	79±14	73±11	70±12
Daily activity, patient	77±17	69±17	66±16	79±13	73±11	68±12
Quality of life, patient	79±17	69±17	68±16	79±14	73±12	70±14

a Wilcoxon signed-ranks test showed that there was a significant (*P*<0.001) decrease in the scores of all items, except haemoptysis, when comparing pretreatment scores with those after two cycles of treatment or after the patient had gone off the study; when all 124 patients were considered together.

b Mann–Whitney test showed there was no difference in the scales between the two arms of treatment either before treatment, two cycles after treatment, or when the patients had gone off the study, except loss of appetite was more severe in the PC arm after two cycles of treatment (patient, *P*=0.021), pain was more severe in the PC arm after two cycles of treatment (patient, *P*=0.022; observer, *P*=0.009) and after the patient had gone off the study (observer, *P*=0.013).

). When considering all the treated patients together, there was a slight, although significant decrease in the scores of all items except haemoptysis (data not shown).

## DISCUSSION

New anticancer drugs in combination with cisplatin usually can prolong patient survival when compared with cisplatin plus conventional anticancer drugs (Le Chevalier *et al*, 1994; Bonomi *et al*, 2000; Baggstrom *et al*, 2002; Raftopoulos *et al*, 2002; Yana *et al*, 2002). However, which new anti-cancer drug is better than the others is still unknown (Scagliotti *et al*, 2002; Schiller *et al*, 2002). Generally, there was no obvious survival difference when these new anticancer drugs were used in combination with cisplatin. However, the toxicity profiles could differ among the new anticancer drugs in combination with cisplatin, due to the pre-existing different toxicity profiles of the new anticancer drugs, such as the higher incidence of peripheral neuropathy with paclitaxel *vs* the more myelosuppressive effect of vinorelbine (Scagliotti *et al*, 2002; Schiller *et al*, 2002). Furthermore, the performance and staging statuses of the study population predicted a major part of the treatment results: the better the performance status and staging status of the study population, the better the response rate and the survival, and also the less severe the treatment toxicities.

Weekly paclitaxel treatment has been found to have a higher response rate and better toxicity profiles in many phase II studies of both chemonaïve patients and patients who have failed previous chemotherapy (Akerley *et al*, 1998; Belani, 2001; Chang *et al*, 2001; Koumakis *et al*, 2002). However, there has been no randomised trial comparing weekly paclitaxel plus cisplatin *vs* other new anticancer drugs plus cisplatin. Thus, we performed this phase II randomised trial. The weekly dose of paclitaxel single-agent treatment given to the patient can be 90 mg m^−2^ or higher (with/without carboplatin or concomitant radiotherapy) (Belani, 2001; Chang *et al*, 2001; Koumakis *et al*, 2002), even up to 175 mg m^−2^ week^−1^ for 6 of 8 weeks (Akerley *et al*, 1998). However, there has been no report of a weekly dose of paclitaxel in combination with cisplatin. The schedule of vinorelbine plus cisplatin used by Le Chevalier et al in the European trial (Le Chevalier *et al*, 1994), with weekly vinorelbine 30 mg m^−2^ plus cisplatin 120 mg m^−2^ on days 1 and 29, and then every 6 weeks, was too toxic to be tolerable in our patients (Perng *et al*, 2000). And so, we initiated a rapid escalation schedule for both paclitaxel and vinorelbine, with dosages from 60 and 20 mg m^−2^ i.v. on days 1, 8, and 15, respectively, while the cisplatin dose was fixed at 60 mg m^−2^ on day 15 of every 4 weeks. Escalation was performed with three patients as a cohort and also within every patient's cycle. It was found very quickly that a dose intensity of paclitaxel 72 mg m^−2^ was less than 66 mg m^−2^ due to frequent dose reductions, and omitting or delaying on day 15; while the dose intensity of vinorelbine was 23 mg m^−2^ higher than 26 mg m^−2^. Thus, it was determined to use paclitaxel 66 mg m^−2^ or vinorelbine 23 mg m^−2^ on days 1, 8, and 15, plus cisplatin 60 mg m^−2^ on day 15 of every 4 weeks. The majority of patients tolerated these two schedules well. For the statistical analysis of treatment efficacy and toxicities, one may argue that the nine pilot patients from each arm should not be included in the analysis. However, all these pilot patients received recommended phase II doses from cycle 2 or 3, and thus we wanted to include them in the statistical analysis of this study, with the intention-to-treat principle. Nevertheless, the overall response rate would be 39.3% in each arm, if we excluded the nine pilot patients from each arm. The median survival was changed to 10.8 and 15.8 months in the PC arm and VC arm, respectively (*P*=0.37); and time to disease progression was 6.1 and 8.8 months, respectively (*P*=0.021), after excluding the nine pilot patients from each arm.

Of course, intrapatient dose escalation for the purpose of assessing the optimal dosage in each patient is probably not the best way to conduct a study. Thus, we did not use intrapatient dose escalation in all the patients. On the other hand, traditional phase I trials sometimes expose too many patients to subtherapeutic doses of the drugs and the trials may take a long time to complete. New trial designs, such as accelerated titration designs, permitting intrapatient dose escalation and using fewer patients per dose level, thus, appear to be effective in reducing the number of patients who are undertreated and speeding the completion of the phase I trial (Simon *et al*, 1997). However, because of the small sample sizes, the optimal recommended doses generally are determined imprecisely, especially when allowing intrapatient dose escalation. Thus, a classical phase I study, followed by a phase II study, probably would be better than the intrapatient dose escalation in the first nine patients, as in our study.

It was found from our study that myelosuppression was the major cause of dose reduction in the VC arm, while severe asthenia and myalgia caused dose reductions in the PC arm. There was no obvious interference in the patient's daily activity if the patient suffered from neutropenia without infection or fever. However, the patients felt discomfort when they had asthenia and myalgia. This kind of discomfort was unpleasant because it occurred ‘weekly’ instead of once every 3 or 4 weeks, which is what usually occurred when paclitaxel was given once every 3 or 4 weeks. This was reflected in our patients’ Lung Cancer Symptom Scale as a more severe loss of appetite in the PC arm after two cycles of treatment. ‘Pain’ was more severe in the PC arm than in the VC arm throughout the whole treatment course. However, the patients’ reporting of their disease severity, daily activity, and life quality showed no difference between the two arms before and through the whole course of treatment (Table 6). Whether it is possible that a lower dose of paclitaxel plus cisplatin than that used in this study could have an equal therapeutic effect and similar or lower toxicities compared to vinorelbine plus cisplatin, is still unknown.

In spite of the fact that patients undergoing VC treatment had a higher incidence of grade 3 or 4 neutropenia than those receiving PC treatment, the duration of neutropenia was short and the recovery quick. Furthermore, vinorelbine is about four times less expensive than paclitaxel in Taiwan. A study performed by the Southwest Oncology Group (SWOG) also showed treatment with paclitaxel plus carboplatin to be substantially and statistically significantly more expensive than treatment with vinorelbine plus cisplatin, especially when considering drug cost (Ramsey *et al*, 2002).

The present study reveals that it is unwise to use weekly paclitaxel plus cisplatin in patients with NSCLC, at least among our Asian population, due to the shorter progression-free survival, relatively more frequent clinic visits, higher drug costs, and higher frequency of asthenia and myalgia which was not reduced after changing the paclitaxel schedule from once every 3 or 4 weeks to once weekly.
